# Respiratory Syncytial Virus Vaccine and Nirsevimab Uptake Among Pregnant People and Their Neonates

**DOI:** 10.1001/jamanetworkopen.2024.60735

**Published:** 2025-02-19

**Authors:** Christine A. Blauvelt, Molly Zeme, Aishwarya Natarajan, Adrienne Epstein, Michelle E. Roh, Amayrani Morales, Nadia Bourdoud, Valerie J. Flaherman, Mary K. Prahl, Stephanie L. Gaw

**Affiliations:** 1 Division of Maternal-Fetal Medicine, Department of Obstetrics, Gynecology, and Reproductive Sciences, University of California, San Francisco; 2 School of Medicine, University of California, San Francisco; 3 Department of Epidemiology and Biostatistics, University of California, San Francisco; 4 Institute for Global Health Sciences, University of California, San Francisco; 5 Department of Pediatrics, University of California, San Francisco; 6 Division of Pediatric Infectious Diseases, Department of Pediatrics, University of California, San Francisco

## Abstract

**Importance:**

Two interventions to prevent severe respiratory syncytial virus (RSV) in infants were approved in 2023—a bivalent prenatal RSV prefusion F protein–based (RSVpreF) vaccine and an infant monoclonal antibody (nirsevimab). Understanding their uptake and clinical outcomes is essential for public health planning.

**Objective:**

To describe uptake of the prenatal RSVpreF vaccine and infant nirsevimab.

**Design, Setting, and Participants:**

This retrospective cohort study was conducted at a single academic center among 647 pregnant individuals eligible for RSVpreF vaccination (32-36 weeks’ gestation between October 15, 2023, and January 31, 2024) and infants eligible for nirsevimab (no prenatal RSVpreF vaccination >14 days before delivery).

**Exposure:**

Pregnancy or birth during the 2023-2024 RSV season.

**Main Outcomes and Measures:**

RSVpreF vaccination among eligible pregnant individuals and nirsevimab administration prior to hospital discharge among eligible infants.

**Results:**

Of 647 eligible pregnant individuals (mean [SD] age, 34.6 [6.2] years; 355 nulliparous [54.9%]; 558 privately insured [86.2%]), 414 (64.0%) received the RSVpreF vaccine. Factors associated with higher RSVpreF uptake included older birthing parent age (adjusted odds ratio [AOR], 1.09; 95% CI, 1.05-1.12), nulliparity (AOR, 1.84; 95% CI, 1.31-2.60), private insurance (AOR, 2.19; 95% CI, 1.27-3.80), non-Hispanic ethnicity (AOR, 2.36; 95% CI 1.57-3.55; reference: Hispanic), receipt of any COVID-19 vaccine (AOR, 7.12; 95% CI, 3.91-13.70), 2023-2024 formula COVID-19 booster vaccine (AOR, 5.62; 95% CI, 3.80-8.48), influenza vaccine (AOR, 8.14; 95% CI, 5.38-12.50), or tetanus-diphtheria-pertussis vaccine (AOR, 6.86; 95% CI, 3.79-13.10). Factors associated with lower RSVpreF uptake included non-English language preference (AOR, 0.24; 95% CI, 0.10-0.52), Black race (AOR, 0.30; 95% CI, 0.16-0.57; reference: Asian), other or unknown race (AOR, 0.48; 95% CI, 0.30-0.76), and multiple gestation (AOR, 0.27; 95% CI, 0.07-0.88). Nirsevimab was administered to 183 of 261 eligible infants (70.1%) prior to hospital discharge. Among those who did not receive RSVpreF or standard prenatal vaccines, 40.4% of their neonates (19 of 47) received nirsevimab; among those who declined infant hepatitis B vaccination, 34.0% of their neonates (17 of 50) received nirsevimab. Respiratory syncytial virus coverage exceeded 80% during all months of the study period except October 2023, the first month during which prenatal RSV vaccination and infant nirsevimab were available. Preterm delivery occurred in 35 of 414 RSVpreF-vaccinated individuals (8.5%) and 43 of 233 unvaccinated individuals (18.5%). In a nested case-control analysis with preterm birth as the outcome, there was no significant association between RSVpreF vaccination and preterm birth (AOR, 1.03; 95% CI, 0.55-1.93).

**Conclusions and Relevance:**

In this cohort study, uptake of the RSVpreF vaccine and infant nirsevimab was high. Nirsevimab uptake was high even among individuals who did not receive routine prenatal or infant vaccines. There was no significant association between RSVpreF vaccination and preterm birth. This study suggests that an RSV prevention strategy that included both prenatal vaccination and infant monoclonal antibody administration had high uptake and reassuring perinatal outcomes.

## Key Points

**Question:**

What was the uptake of the bivalent respiratory syncytial virus (RSV) prefusion F protein–based (RSVpreF) vaccine during pregnancy and of infant monoclonal antibody against RSV (nirsevimab) during the introductory season?

**Findings:**

In this cohort study of 647 pregnant individuals, 64.0% of eligible pregnant individuals received the RSVpreF vaccine and 70.1% of eligible neonates received nirsevimab, which translated to more than 80% coverage against RSV during all but the first month of the study period. There was no significant association between RSVpreF vaccination and preterm birth in a nested case-control study.

**Meaning:**

This study suggests that an RSV prevention strategy that included both prenatal vaccination and infant monoclonal antibody administration had high uptake and reassuring perinatal outcomes.

## Teaser

This cohort study describes uptake of the prenatal RSVpreF vaccine and of infant monoclonal antibody nirsevimab for prevention of severe respiratory syncytial virus during the first season they were available.

## Introduction

Respiratory syncytial virus (RSV) is the leading cause of infant hospitalization in the US, with up to 2% of infants younger than 6 months hospitalized for RSV each year.^1,2,3^ Globally, RSV accounts for 6.7% of deaths among children younger than 12 months.^4^ In 2023, the US Food and Drug Administration (FDA) approved 2 new interventions to prevent severe RSV disease among infants during the first 6 months of life: a prenatal vaccine and an infant monoclonal antibody. The bivalent RSV prefusion F protein–based (RSVpreF) vaccine is given to pregnant individuals and protects infants through maternal-fetal antibody transfer.^5^ Nirsevimab, a monoclonal antibody active against the RSV F protein, is administered to infants younger than 8 months if their birth parent did not receive the RSVpreF vaccine at least 2 weeks before birth.^6,7,8^ Nirsevimab has a half-life of 2 months, enabling only 1 administration for coverage of an entire RSV season.^9^

Although there were no statistically significant safety signals from the RSVpreF vaccine clinical trial, there was a higher rate of preterm birth in the vaccine group (5.6% vs 4.7%).^5^ This finding is of particular interest because a phase 3 trial of another candidate maternal prefusion F protein–based vaccine (RSVpreF-Mat) was terminated due to significantly higher rates of preterm birth in the vaccine group (6.8% vs 4.9%; relative risk, 1.37; 95% CI, 1.08-1.74).^10^ Existing research has not identified a mechanism for the increased rates of preterm birth, although most cases in the phase 3 RSVpreF vaccine trial occurred in low-income and middle-income countries during August to December 2021, coinciding with a wave of B.1.617.2 (Delta) variant dominant COVID-19 disease.^10^ Due to the possible association between the RSVpreF vaccine and preterm birth, the FDA limited its approval of the RSVpreF vaccine to administration between 32 and 36 weeks’ gestation.^11^ When results from the RSVpreF vaccine phase 3 clinical trial were assessed for the approved 32-week to 36-week window, rates of preterm birth were similar in the vaccine and placebo groups (4.2% vs 3.7%).^12^ However, there are limited data regarding outcomes in routine clinical practice, particularly preterm birth rates, after RSVpreF vaccination.^13^

It is crucial to understand the uptake and outcomes of the newly approved pharmacologic interventions against infant RSV to effectively plan public health initiatives. In this study, we describe the uptake of the RSVpreF vaccine among eligible pregnant people and nirsevimab among eligible infants during the first season of availability. We also describe obstetric outcomes between those who received the RSVpreF vaccine and those who did not.

## Methods

The University of California, San Francisco (UCSF) institutional review board approved this study with a waiver of informed consent because of the retrospective design and use of deidentified data. The Strengthening the Reporting of Observational Studies in Epidemiology (STROBE) reporting guideline was used when preparing the manuscript.^14^

### Study Population

We performed a retrospective cohort study of individuals who received prenatal care and delivered at UCSF. We queried all deliveries from October 15, 2023, through April 15, 2024. Individuals were included if they were 32 to 36 weeks’ gestation from October 15, 2023, through January 31, 2024, which is the period when the RSVpreF vaccine was readily available in San Francisco commercial pharmacies or at our institution. Individuals were excluded if they delivered prior to 32 weeks’ gestation or if they did not receive their prenatal care at UCSF.

### Variables and Outcome Measures

We queried the California Department of Public Health, California Immunization Registry (CAIR2) to obtain vaccination records.^15^ Vaccines of interest included the RSVpreF vaccine during pregnancy, any COVID-19 vaccine including those prior to pregnancy, the COVID-19 2023-2024 formulation booster during pregnancy, the influenza vaccine during pregnancy, and the tetanus-diphtheria-pertussis (Tdap) vaccine during pregnancy. We also queried medical records for nirsevimab administration among eligible infants during their birth hospitalization. Nirsevimab became available at our institution on October 11, 2023, and all eligible newborns were offered this medication prior to hospital discharge. Liveborn neonates of those who did not receive the RSVpreF vaccine at least 14 days prior to delivery were eligible for nirsevimab.

Baseline characteristics obtained from pregnant patients’ medical records included birthing parent age at delivery, parity, race and ethnicity, primary insurance payer, and language preference. Race and ethnicity classifications were obtained from the electronic health record, which are typically self-reported by patients during hospital registration. Race and ethnicity were included in this study because members of racial and ethnic minority groups may experience disparities in vaccine uptake.

We assessed birthing parent medical conditions, including asthma, other pulmonary disease (eg, cystic fibrosis and chronic obstructive pulmonary disease), chronic hypertension, other cardiovascular disease (eg, congenital heart disease and arrhythmias), pregestational diabetes, gestational diabetes, obesity, multiple gestation, and use of assisted reproductive technologies in the current pregnancy. Pregnancy-specific clinical outcomes included gestational age at delivery, preterm delivery, preterm labor, preterm premature rupture of membranes, fetal growth restriction, early fetal growth restriction (diagnosed prior to 32 weeks’ gestation), pregnancy-induced hypertension, and oligohydramnios. Labor and delivery outcomes included chorioamnionitis, cesarean delivery, and postpartum hemorrhage.

Neonatal outcomes included birth weight, neonatal intensive care unit (NICU) admission (including admission for observation with subsequent transfer to the well-baby nursery), and stillbirth. At our institution, low-risk infants with elevated bilirubin needing phototherapy are admitted to the NICU by hospital policy. We also queried medical records for neonatal receipt of the hepatitis B vaccine, intramuscular vitamin K, and erythromycin ophthalmic ointment prior to hospital discharge. Definitions for variables and outcome measures are included in eTable 1 in Supplement 1.

### Statistical Analysis

The primary outcomes were RSVpreF vaccination among eligible pregnant individuals and nirsevimab administration among eligible infants prior to hospital discharge. Descriptive analyses were performed for all patient characteristics and clinical variables. Between-group differences were assessed by the χ^2^ test for categorical variables and by the *t* test for continuous variables. Univariable and multivariable binary logistic regression models were used to quantify the association between patient characteristics and receipt of the prenatal RSVpreF vaccine or infant nirsevimab. Unadjusted odds ratios (ORs), adjusted ORs (AORs), and 95% CIs were reported. For adjusted estimates, directed acyclic graphs (eFigures 2 and 3 in Supplement 1) were used to represent our assumptions about the plausible causal structure and to guide covariate selection for multivariable models. This approach ensured that only common causes of the risk factor and outcome were included as adjustment covariates, avoiding bias from adjusting for possible mediators or colliders.^16^

We conducted a nested case-control analysis to further clarify the association between RSVpreF vaccination and preterm birth. Cases were defined as pregnancies that resulted in a preterm birth (<37 weeks) and controls were defined as pregnancies that resulted in a term birth (≥37 weeks). Cases and controls were matched on estimated due date within 14 days to account for seasonal changes in the rollout of the RSVpreF vaccine and any seasonality in preterm birth. To maximize use of the available data, cases were matched with up to 7 controls. After matching, RSVpreF vaccination status was set to unvaccinated for any control who received the vaccine after the gestational week when the control’s matched case delivered. This step was taken to ensure that cases and their corresponding controls were permitted the same amount of time that they were eligible to be vaccinated. A multivariable conditional logistic regression model was used to assess the association between RSVpreF vaccination and preterm birth. The model included birthing parent age at delivery, parity, race and ethnicity, primary insurance payer, birthing parent cardiovascular disease, birthing parent pregestational diabetes, multiple gestation, use of assisted reproductive technologies in the current pregnancy, early fetal growth restriction (diagnosed at <32 weeks’ gestation), and receipt of the Tdap vaccine during pregnancy. These covariates were selected as they may be associated with both the likelihood of receiving the RSVpreF vaccine and the risk of preterm birth. Adjusted odds ratios and 95% CIs were reported.

All statistical tests were 2-tailed, and *P* < .05 was considered statistically significant. Statistical analyses were performed using R, version 4.3.3 (R Project for Statistical Computing), Python, version 3.8.5 (Python Software Foundation), SciPy, version 1.5.2 (The SciPy community), and pandas, version 1.1.2 (Python Software Foundation).

## Results

There were 1182 individuals who delivered between October 15, 2023, and April 15, 2024. Among these, 652 individuals were eligible for RSVpreF vaccination, defined as being 32 to 36 weeks’ gestation between October 15, 2023, and January 31, 2024. Five patients (0.8%) were excluded for having received their prenatal care elsewhere. The remaining 647 individuals (mean [SD] age, 34.6 [6.2] years; 355 nulliparous [54.9%]; 558 privately insured [86.2%]; 174 Asian individuals [26.9%]; 52 Black individuals [8.0%]; 118 Hispanic individuals [18.2%]; 250 White individuals [38.6%]; 32 individuals of ≥2 races [4.9%]; and 141 individuals of other [self-reported other race, Native American and Alaskan Native, and Native Hawaiian or Pacific Islander] or unknown race [21.8%]) were included in the final analysis (eFigure 1 and eTable 2 in Supplement 1). Of these, 414 (64.0%) received the RSVpreF vaccine during pregnancy. RSVpreF vaccines were administered at hospital clinics (77.5% [321 of 414]) and private pharmacies (21.3% [88 of 414]) at a mean (SD) gestational age of 33.9 (1.5) weeks. Most RSVpreF vaccines (92.8% [384 of 414]) were administered at least 14 days prior to delivery.

Of the 647 pregnant people meeting the inclusion criteria, 30 (4.6%) were vaccinated within 14 days of delivery, and 233 (36.0%) did not receive a prenatal RSVpreF vaccine. Two infants (0.3%) were stillborn and therefore ineligible for nirsevimab. This left 261 infants who were eligible for nirsevimab (eFigure 1 in Supplement 1). There were significant differences between infants eligible and ineligible for nirsevimab, including earlier mean (SD) gestational age at delivery (38.2 [2.1] vs 39.1 [1.3] weeks; *P* < .001) and higher rates of NICU admission (62 of 261 [23.8%] vs 37 of 386 [9.6%]; *P* < .001) (Table 1). In addition, the birthing parents of those who were eligible for nirsevimab had higher rates of preterm birth (54 of 261 [20.7%] vs 24 of 386 [6.2%]; *P* < .001), preterm labor (12 of 261 [4.6%] vs 4 of 386 [1.0%]; *P* = .009), preterm premature rupture of membranes (15 of 261 [5.7%] vs 9 of 386 [2.3%]; *P* = .04), and pregnancy-induced hypertension (85 of 261 [32.6%] vs 85 of 386 [22.0%]; *P* = .003). Among the 261 neonates eligible for nirsevimab, 183 (70.1%) received nirsevimab prior to hospital discharge. Among the cohort of pregnant individuals who received neither the RSVpreF vaccine nor any standard prenatal vaccines (influenza or Tdap) during pregnancy, 40.4% of their neonates (19 of 47) received nirsevimab. In addition, among infants who did not receive the hepatitis B vaccine, erythromycin eye ointment, or intramuscular vitamin K prior to hospital discharge, 34.0% (17 of 50), 31.0% (9 of 29), and 0.0% (0 of 8), respectively, received nirsevimab.

**Table 1.** Patient Characteristics Between Neonates Eligible and Those Ineligible for Nirsevimab^a^

Outcome	No. (%)		*P* value
	Eligible (n = 261)	Ineligible (n = 386)	
Gestational age at delivery, mean (SD), wk	38.2 (2.1)	39.1 (1.3)	<.001
Preterm delivery	54 (20.7)	24 (6.2)	<.001
Preterm labor	12 (4.6)	4 (1.0)	.009
Preterm premature rupture of membranes	15 (5.7)	9 (2.3)	.04
Fetal growth restriction	15 (5.7)	25 (6.5)	.83
Pregnancy-induced hypertension	85 (32.6)	85 (22.0)	.003
Oligohydramnios	5 (1.9)	13 (3.4)	.39
NICU admission	62 (23.8)	37 (9.6)	<.001

Abbreviation: NICU, neonatal intensive care unit.

^a^ Liveborn neonates of those who did not receive the bivalent respiratory syncytial virus prefusion F protein vaccine at least 14 days prior to delivery were eligible for nirsevimab.

**Figure 1.**
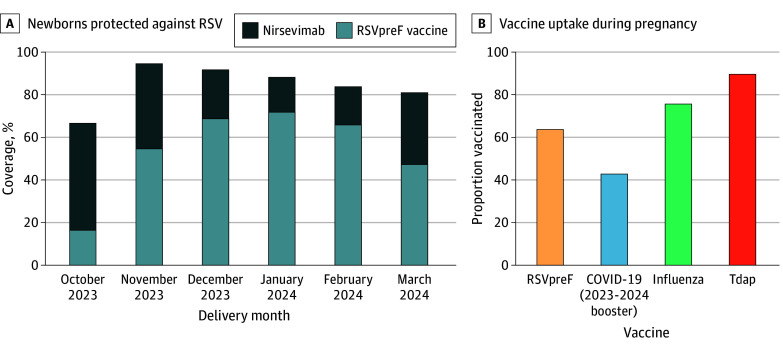
Uptake of Prenatal Vaccines and Infant Nirsevimab A, Monthly proportion of newborns during the study period who were protected against respiratory syncytial virus (RSV), either through bivalent RSV prefusion F protein (RSVpreF) vaccination administered at least 14 days before delivery or infant nirsevimab administration prior to hospital discharge. B, Uptake of vaccines during pregnancy. Tdap indicates tetanus-diphtheria-pertussis.

Figure 1A shows the monthly proportion of newborns delivered during the study period who received protection against RSV either through prenatal RSVpreF vaccination at least 14 days prior to delivery or infant nirsevimab administration prior to hospital discharge. Respiratory syncytial virus coverage exceeded 80% during all months of the study period except October 2023, the first month during which prenatal RSVpreF vaccination and infant nirsevimab were available. Figure 1B shows the proportion of individuals receiving other vaccinations during pregnancy, with 43.0% (278 of 647) receiving the 2023-2024 formula COVID-19 booster, 75.9% (491 of 647) receiving the influenza vaccine, and 89.8% (581 of 647) receiving the Tdap vaccine.

**Figure 2.**
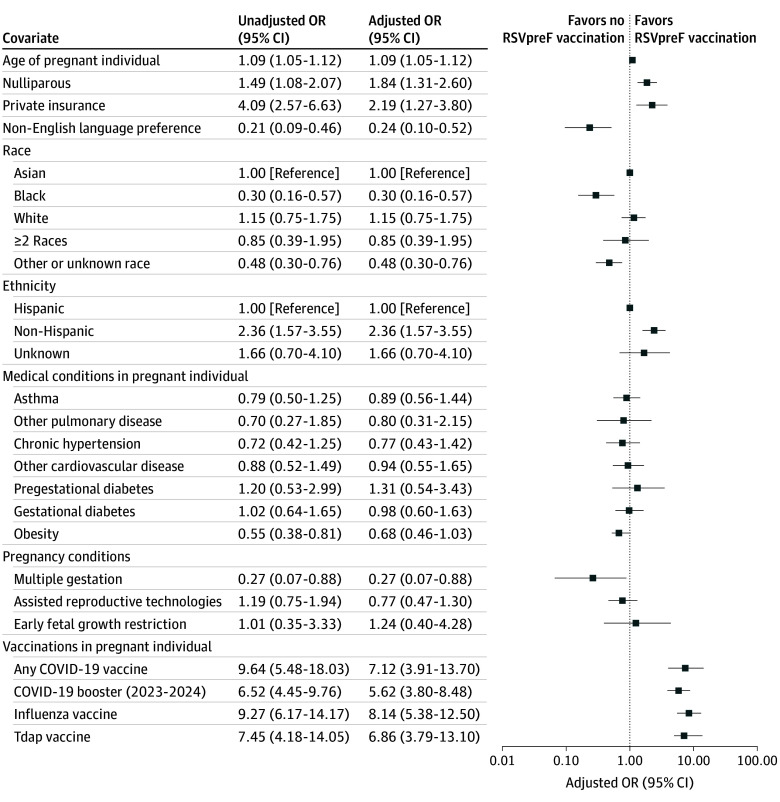
Univariable and Multivariable Logistic Regression Analyses for Characteristics Associated With Bivalent Respiratory Syncytial Virus Prefusion F Protein (RSVpreF) Vaccination Among Eligible Pregnant Individuals For adjusted estimates, a directed acyclic graph (eFigure 2 in Supplement 1) was used to represent our assumptions of the causal structure and to guide covariate selection for adjustment, ensuring that only those necessary to close backdoor paths between the risk factor of interest and the outcome were included. Race and ethnicity data were obtained from the electronic medical record. The “other” race category included self-reported other race, Native American or Alaska Native, and Native Hawaiian or Pacific Islander. Early fetal growth restriction includes cases diagnosed prior to 32 weeks’ gestation. The odds ratio (OR) for age represents the change in odds of RSVpreF vaccination associated with each 1-year increase in age. Tdap indicates tetanus-diphtheria-pertussis.

Univariable and multivariable binary logistic regression models were used to examine the association between prenatal RSVpreF vaccination and clinical characteristics (Figure 2; eTable 2 in Supplement 1). Factors associated with higher RSVpreF vaccine uptake included older birthing parent age (AOR, 1.09; 95% CI, 1.05-1.12), nulliparity (AOR, 1.84; 95% CI, 1.31-2.60), private insurance (AOR, 2.19; 95% CI, 1.27-3.80), non-Hispanic ethnicity (AOR, 2.36; 95% CI, 1.57-3.55; reference: Hispanic), receipt of any COVID-19 vaccine (AOR, 7.12; 95% CI, 3.91-13.70), 2023-2024 formula COVID-19 booster vaccine (AOR, 5.62; 95% CI, 3.80-8.48), influenza vaccine (AOR, 8.14; 95% CI, 5.38-12.50), or Tdap vaccine (AOR, 6.86; 95% CI, 3.79-13.10). Factors associated with lower RSVpreF vaccine uptake included non-English language preference (AOR, 0.24; 95% CI, 0.10-0.52), Black race (AOR, 0.30; 95% CI, 0.16-0.57; reference: Asian), other or unknown race (AOR, 0.48; 95% CI, 0.30-0.76), and multiple gestation (AOR, 0.27; 95% CI, 0.07-0.88).

**Figure 3.**
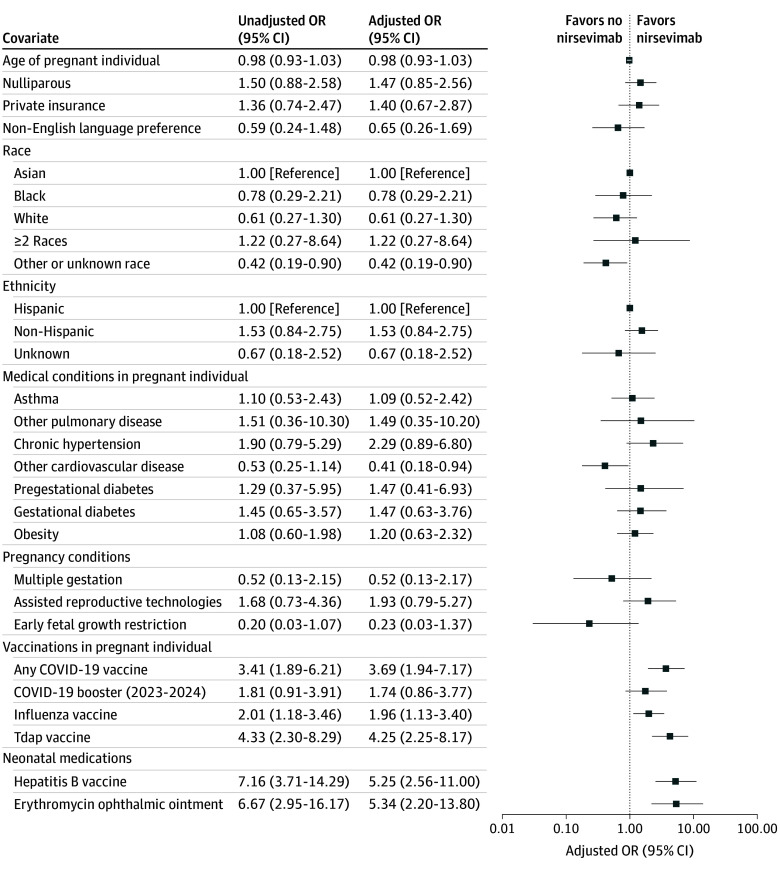
Univariable and Multivariable Logistic Regression Analyses for Characteristics Associated With Neonatal Nirsevimab Administration Prior to Hospital Discharge For adjusted estimates, a directed acyclic graph (eFigure 3 in Supplement 1) was used to represent our assumptions of the causal structure and to guide covariate selection for adjustment, ensuring that only those necessary to close backdoor paths between the risk factor of interest and the outcome were included. Race and ethnicity data were obtained from the electronic medical record. The “other” race category included self-reported other race, Native American or Alaska Native, and Native Hawaiian or Pacific Islander. Early fetal growth restriction includes cases diagnosed prior to 32 weeks’ gestation. The odds ratio (OR) for age represents the change in odds of nirsevimab administration associated with each 1-year increase in birthing parent age. Tdap indicates tetanus-diphtheria-pertussis.

Univariable and multivariable binary logistic regression models were also used to examine the association between patient characteristics and nirsevimab administration prior to hospital discharge among eligible neonates (Figure 3; eTable 3 in Supplement 1). Factors associated with neonatal nirsevimab administration in the multivariable logistic regression analysis included higher rates of birthing parent COVID-19 vaccination (AOR, 3.69; 95% CI, 1.94-7.17), higher rates of birthing parent influenza vaccination (AOR, 1.96; 95% CI, 1.13-3.40), higher rates of birthing parent Tdap vaccination (AOR, 4.25; 95% CI, 2.25-8.17), higher rates of neonatal hepatitis B vaccination (AOR, 5.25; 95% CI, 2.56-11.00), and higher rates of use of neonatal erythromycin ophthalmic ointment (AOR, 5.34; 95% CI, 2.20-13.80). Factors associated with lower neonatal nirsevimab uptake included other or unknown race (AOR, 0.42; 95% CI, 0.19-0.90) and lower rates of birthing parent cardiovascular disease (AOR, 0.41; 95% CI, 0.18-0.94). Neonates who received nirsevimab were significantly more likely to receive intramuscular vitamin K than those who did not receive nirsevimab (183 [100.0%] vs 70 of 78 [89.7%]; *P* < .001). Intramuscular vitamin K was not included in the univariable or multivariable logistic regression models because intramuscular vitamin K was received by all individuals in the nirsevimab group, leading to complete separation and precluding reliable estimation of its association with outcomes.

Table 2 shows the crude prevalences of obstetric outcomes between individuals who did and individuals who did not receive the RSVpreF vaccine during pregnancy. Vaccinated individuals delivered at a later mean (SD) gestational age (39.0 [1.4] vs 38.4 [2.1] weeks; *P* < .001) and had lower rates of preterm delivery (35 of 414 [8.5%] vs 43 of 233 [18.5%]; *P* < .001). The mean (SD) gestational age at delivery for those who delivered preterm was lower among the unvaccinated group than the vaccinated group (34.7 [1.3] vs 35.8 [1.0] weeks; *P* < .001). The mean (SD) interval between RSVpreF vaccination and delivery was longer for those who delivered at full term than those who delivered preterm (34.4 [13.0] vs 17.1 [8.4] days; *P* < .001). Neonates of vaccinated individuals had higher mean (SD) birth weights (3289 [517] vs 3150 [601] g; *P* = .003) and were less likely to be admitted to the NICU (46 of 414 [11.1%] vs 53 of 233 [22.8%]; *P* < .001).

**Table 2.** Clinical Outcomes Associated With Uptake of the RSVpreF Vaccine During Pregnancy

Outcome	No. (%)		*P* value
	Vaccinated (n = 414)	Unvaccinated (n = 233)	
Gestational age at delivery, mean (SD), wk	39.0 (1.4)	38.4 (2.1)	<.001
Preterm delivery	35 (8.5)	43 (18.5)	<.001
Preterm labor	9 (2.2)	7 (3.0)	.70
Preterm premature rupture of membranes	13 (3.1)	11 (4.7)	.42
Fetal growth restriction	27 (6.5)	13 (5.6)	.76
Pregnancy-induced hypertension	99 (23.9)	71 (30.5)	.08
Oligohydramnios	13 (3.1)	5 (2.2)	.62
Chorioamnionitis	47 (11.4)	26 (11.2)	>.99
Cesarean delivery	135 (32.6)	64 (27.5)	.20
Postpartum hemorrhage	83 (20.0)	38 (16.3)	.29
Birth weight, mean (SD), g	3289 (517)	3150 (601)	.003
NICU admission	46 (11.1)	53 (22.8)	<.001
Stillbirth	0	2 (0.86)	.25

Abbreviations: NICU, neonatal intensive care unit; RSVpreF, bivalent respiratory syncytial virus prefusion F protein.

We conducted a nested case-control analysis to further clarify the association between RSVpreF vaccination and preterm birth. We matched 75 cases who delivered preterm with 519 controls who delivered at term, with matching performed based on estimated due date within 14 days. After matching, any controls who received RSVpreF vaccination after the delivery date of their matched case were classified as unvaccinated (n = 123). In a multivariable conditional logistic regression model adjusting for relevant covariates, there was no significant association between preterm birth and RSVpreF vaccination (AOR, 1.03; 95% CI, 0.55-1.93) (eTable 4 in Supplement 1).

## Discussion

We report high uptake of the RSVpreF vaccine and infant monoclonal antibody nirsevimab during the introductory season, with 64.0% of eligible pregnant individuals and 70.1% of eligible neonates at our institution receiving these interventions. Infant coverage against RSV, through either birthing parent vaccination or infant monoclonal antibody administration, was high and exceeded 80% in all but the first month of the study period. A total of 40.4% of birth parents who declined RSVpreF and standard prenatal vaccines (Tdap and influenza) during pregnancy accepted nirsevimab for their infants. Furthermore, 34.0% of eligible infants who did not receive the hepatitis B vaccine received nirsevimab. A case-control analysis found that RSVpreF vaccination was not associated with increased risk of preterm birth.

The uptake of the RSVpreF vaccine in our population was rapid and high: in December 2023, nearly 70% of birthing parents were vaccinated, corresponding with quick uptake of vaccination after rollout. In comparison, data from the Centers for Disease Control and Prevention (CDC) Vaccine Safety Datalink showed only 17.8% RSV vaccine coverage among pregnant individuals nationally by January 31, 2024.^17^ We have considered several reasons for the high uptake of the RSVpreF vaccine at our institution. Most importantly, the vaccine was available in our prenatal clinics and offered to all eligible pregnant individuals. There was widespread marketing around the RSVpreF vaccine for older adults, which began months before the vaccine was approved for pregnancy and may have increased awareness and uptake of the RSVpreF vaccine.^18^ The severity of the previous (2022-2023) RSV season may have also increased the public’s awareness of RSV disease and made parents more willing to accept preventive measures for their infants.^19^ Furthermore, the peptide-based technology behind the RSVpreF vaccine may have been more acceptable to patients compared with the novel mRNA delivery platform of the COVID-19 vaccines.

Nirsevimab uptake was also high, with 70% of eligible neonates receiving the monoclonal antibody prior to hospital discharge. The high uptake of nirsevimab among our population may be due to the convenience of monoclonal antibody administration. Nirsevimab was available in our hospital and offered to all eligible newborns prior to hospital discharge. This availability reduced logistical barriers, as parents did not have to go to any secondary locations or make additional visits for their infants to receive nirsevimab. In contrast, the RSVpreF vaccine was difficult to find in local pharmacies when it was first approved, and it had a very narrow window of administration eligibility (32-36 weeks’ gestation). In addition, our hospital did not apply extra charges to receive nirsevimab, irrespective of the patient’s insurance status, which reduced financial barriers to accessing nirsevimab. Finally, a pregnancy intervention may have been perceived as higher risk than an infant intervention, particularly in the context of early reports of a potential for increased preterm birth rates in the RSVpreF and RSVpreF-Mat clinical trials.^10^

Even within the groups that declined standard prenatal vaccines and the neonatal hepatitis B vaccine, uptake of nirsevimab was still high, at 40% and 34%, respectively. Individuals may view monoclonal antibodies differently than vaccines, which may account for increased uptake of nirsevimab among patients who tend to decline vaccines. Some may view passive immunization as lower risk than active immunization. Finally, there is widespread misinformation regarding vaccines in general, which may contribute to hesitancy, particularly toward a new vaccine.^20^ This effect has been well demonstrated with other vaccines, such as the COVID-19 vaccine.^21^

Our study did not reveal any safety concerns from RSVpreF vaccination. There were concerns about increased risk of preterm birth after the phase 3 RSVpreF and RSVpreF-Mat trials. Our study of clinical outcomes after RSVpreF vaccination from 32 to 36 weeks’ gestation did not confirm this finding, although it is possible that our case-control analysis may not have had sufficient statistical power to identify a difference similar to that noted in the phase 3 trials, in part because the FDA approval of the vaccine was from only 32 to 36 weeks’ gestation. A recent study of 2 New York City hospitals similarly found no association between RSVpreF vaccination and increased risk for preterm birth or other adverse perinatal outcomes.^13^ The crude prevalence of preterm birth in our cohort was significantly lower in the vaccinated group (8.5% vs 18.5%; *P* < .001). Preterm delivery rates in the vaccinated group were similar to historical preterm birth rates in San Francisco and at our institution.^22,23^ We hypothesize that the higher preterm birth rate among unvaccinated individuals was in part due to logistical challenges of administering the vaccine within a narrow, 1-month window of pregnancy, as well as the requirement to be 32 weeks’ gestation or higher. People who delivered preterm had less time to get vaccinated than those who delivered at term. Our findings provide reassurance as to the RSVpreF vaccine’s safety and support its potential as a valuable tool in infant RSV prevention.

Our study showed that a 2-pronged approach with both prenatal RSVpreF vaccination and infant nirsevimab enabled high coverage against RSV. During the 2023-2024 season, there were significant nationwide shortages of nirsevimab, related to high costs of the medication, demand outpacing supply, and logistical barriers with distribution.^24^ Although the Advisory Committee on Immunization Practices initially recommended administration for infants younger than 8 months, these shortages led the CDC to recommend nirsevimab only for infants younger than 6 months.^24^ Prioritization of prenatal vaccination may relieve the financial and supply-related strains of nirsevimab. Uptake of the RSVpreF vaccine was high in our cohort, and while many unvaccinated individuals delivered prematurely, based on our case-control analyses, this was unlikely due to a causal mechanism but rather because those who delivered prematurely had a shorter time frame during which they could receive the vaccine, even if they intended to do so. Earlier vaccination prior to 32 weeks’ gestation could be considered for individuals with planned preterm delivery, such as those admitted for preeclampsia with planned delivery at 34 weeks. This would provide protection for preterm infants who are at the highest risk of severe RSV.

### Strengths and Limitations

Our study has some strengths, including providing observational data on uptake of the prenatal RSVpreF vaccine and infant nirsevimab, as well as obstetric outcomes after receipt of the RSVpreF vaccine. Vaccination records were obtained through the California Department of Public Health, California Immunization Registry, allowing us to capture immunizations given at both our institution and outside pharmacies. Detailed medical record review allowed us to have complete and accurate clinical data, including granular data not captured by most population-wide datasets (eg, infant intramuscular vitamin K and other medications).

Several limitations of our study should be acknowledged. These include the retrospective nature of data collection at a single institution. Generalizability may be limited to similar populations, particularly those who received the vaccine after 32 weeks’ gestation. Finally, there is potential for unmeasured confounding of our risk factor and case-control analyses.

Future work should continue to evaluate the safety and efficacy of the RSVpreF vaccine and nirsevimab in preventing infant RSV disease, as data outside of clinical trial settings are limited. There have been no head-to-head comparisons of RSVpreF vaccination vs nirsevimab for protection against RSV disease. Qualitative studies are essential to better understand reasons for uptake and declination of the 2 interventions. Prior work into vitamin K and hepatitis B vaccine refusal has identified several themes, including perception of risk, desire to avoid neonatal injections in the immediate postnatal period, concerns about safety of the medications, and general mistrust of the medical system and pharmaceutical companies.^25,26,27^ It is unknown whether these concerns similarly apply to the RSVpreF vaccine and nirsevimab.

## Conclusions

In this cohort study of pregnant individuals eligible for RSV vaccination during the 2023-2024 season, uptake of the RSVpreF vaccine and infant nirsevimab during the introductory RSV season was high, providing more than 80% coverage at our institution. Nirvesimab was accepted even among individuals who declined other routine prenatal or infant vaccines. There was no significant association between RSVpreF vaccination and preterm birth. This study suggests that an RSV prevention strategy that included both prenatal vaccination and infant monoclonal antibody administration had high uptake and reassuring perinatal outcomes.

## Notes

Supplement 1. **eFigure 1.** Flow Chart of Included Patients **eTable 1.** Definitions for Variables and Outcome Measures **eFigure 2.** Directed Acyclic Graph for RSVpreF Vaccination **eFigure 3.** Directed Acyclic Graph for Nirsevimab **eTable 2.** Characteristics Associated With RSVpreF Vaccination **eTable 3.** Characteristics Associated With Neonatal Nirsevimab Administration Prior to Hospital Discharge **eTable 4.** Multivariable Conditional Logistic Regression Analysis for Nested Case-Control Analysis With Preterm Birth as the Primary Outcome

Supplement 2.
Data Sharing Statement
