# Supplementary material for: Respiratory Syncytial Virus Vaccine and Nirsevimab Uptake Among Pregnant People and Their Neonates
Source: JAMA Netw Open. 2025 Feb 19;8(2):e2460735. doi: 10.1001/jamanetworkopen.2024.60735 (PMC11840647; doi:10.1001/jamanetworkopen.2024.60735)
Supplement: Supplement 1. — eFigure 1. Flow Chart of Included Patients eTable 1. Definitions for Variables and Outcome Measures eFigure 2. Directed Acyclic Graph for RSVpreF Vaccination eFigure 3. Directed Acyclic Graph for Nirsevimab eTable 2. Characteristics Associated With RSVpreF Vaccination eTable 3. Characteristics Associated With Neonatal Nirsevimab Administration Prior to Hospital Discharge eTable 4. Multivariable Conditional Logistic Regression Analysis for Nested Case-Control Analysis With Preterm Birth as the Primary Outcome [file jamanetwopen-e2460735-s001.pdf]

Supplementary Online Content

Blauvelt CA, Zeme M, Natarajan A, et al. Respiratory syncytial virus vaccine and nirsevimab uptake among pregnant people and their neonates. *JAMA Netw Open.* 2025;8(2):e2460735. doi:10.1001/jamanetworkopen.2024.60735

eFigure 1. Flow Chart of Included Patients
eTable 1. Definitions for Variables and Outcome Measures
eFigure 2. Directed Acyclic Graph for RSVpreF Vaccination
eFigure 3. Directed Acyclic Graph for Nirsevimab
eTable 2. Characteristics Associated With RSVpreF Vaccination
eTable 3. Characteristics Associated With Neonatal Nirsevimab Administration Prior to Hospital Discharge
eTable 4. Multivariable Conditional Logistic Regression Analysis for Nested Case-Control Analysis With Preterm Birth as the Primary Outcome

This supplementary material has been provided by the authors to give readers additional information about their work.

© 2025 Blauvelt CA et al. *JAMA Network Open.*

eFigure 1. Flow Chart of Included Patients

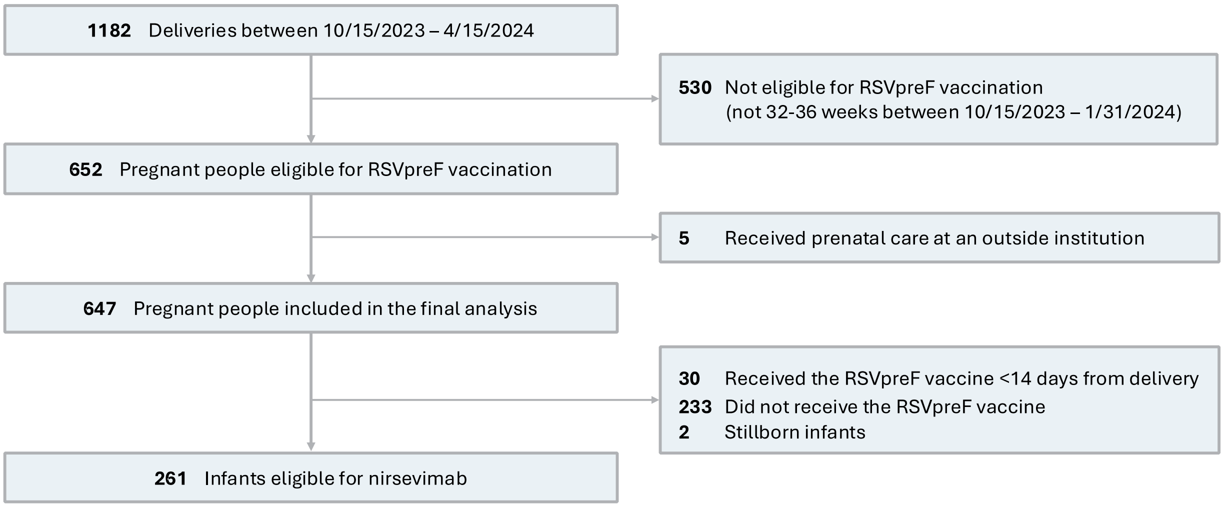

© 2025 Blauvelt CA et al. *JAMA Network Open.*

eTable 1. Definitions for Variables and Outcome Measures

**Variable/Outcome**	**Definition**
Baseline characteristics	
Race	Race categories included Asian, Black, White, Two or more races, Other, and Unknown race. The “other” race category included self-reported Other race, Native American and Alaskan Native, and Native Hawaiian or Pacific Islander. Individuals with more than one race category in the electronic medical record were classified as “Two or more races.”
Maternal medical conditions	
Pregestational diabetes	Diabetes diagnosed prior to pregnancy or hemoglobin A1C ≥6.5% prior to 14 weeks’ gestation
Gestational diabetes	Abnormal glucose tolerance testing after 14 weeks’ gestation
Obesity	Patient-reported BMI prior to pregnancy or earliest documented BMI during pregnancy ≥30 kg/m^2
Pregnancy-specific outcomes	
Preterm delivery	Delivery at less than 37 weeks’ gestation
Preterm labor	Uterine contractions leading to cervical change at less than 37 weeks’ gestation
Preterm premature rupture of membranes	Rupture of membranes occurring at less than 37 weeks’ gestation
Fetal growth restriction	Sonographic estimated fetal weight or abdominal circumference less than the 10th percentile for gestational age
Early fetal growth restriction	Fetal growth restriction diagnosed at less than 32 weeks’ gestation
Pregnancy-induced hypertension	New elevated blood pressure of 140/90mmHg or higher after 20 weeks’ gestation, or superimposed preeclampsia in a patient with chronic hypertension
Oligohydramnios	Deepest vertical pocket of amniotic fluid on ultrasound less than 2cm
Labor and delivery outcomes	
Chorioamnionitis	Intrapartum fever and fetal tachycardia, leukocytosis >15,000 per microliter, or purulent or foul-smelling amniotic fluid
Postpartum hemorrhage	Delivery blood loss greater than 1000mL

© 2025 Blauvelt CA et al. *JAMA Network Open.*

eFigure 2. Directed Acyclic Graph for RSVpreF Vaccination

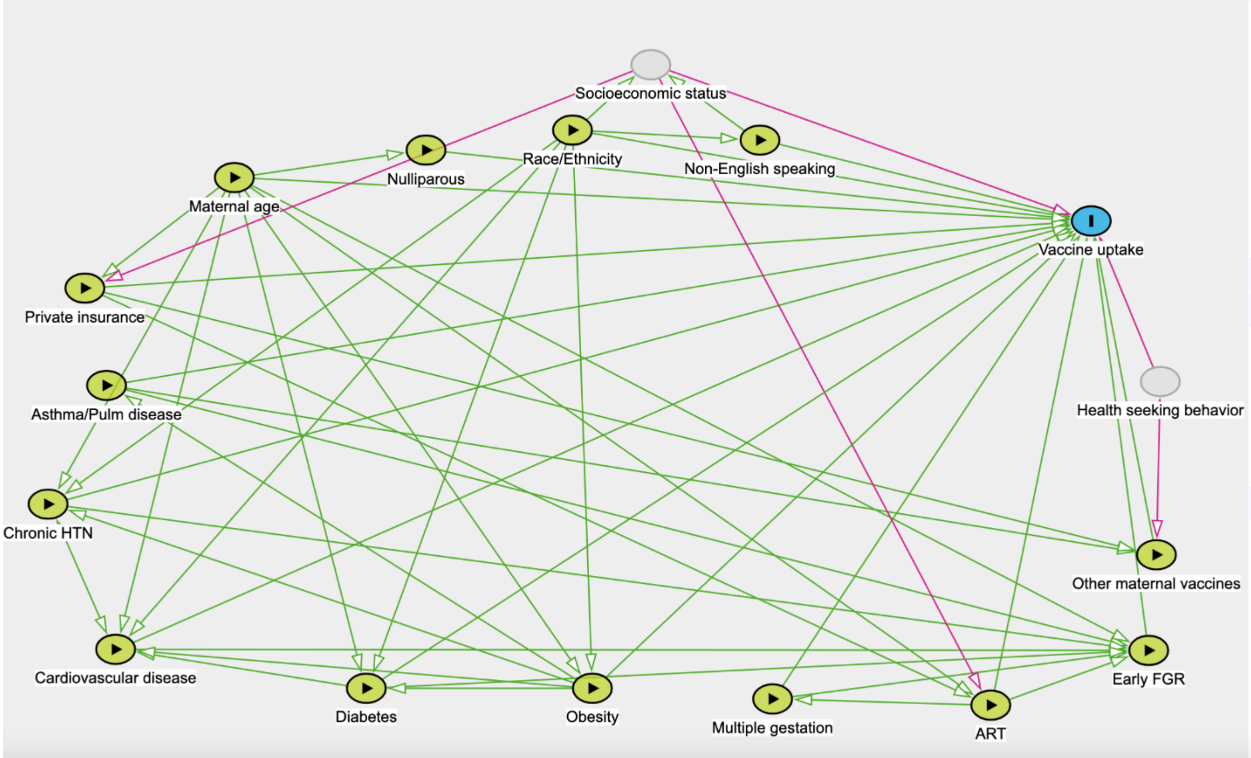

Covariate of interest	Secondary adjusted covariates
Age	None
Nulliparous	Age
Private insurance	Age, race, non-English language preference
Non-English language preference	Race
Race	None
Ethnicity	None
Asthma	Obesity
Other pulmonary disease	Obesity
Chronic hypertension	Age, race, diabetes, obesity, cardiovascular disease
Other cardiovascular disease	Age, race, chronic hypertension, diabetes, obesity
Pregestational diabetes	Age, race, obesity
Gestational diabetes	Age, race, obesity
Obesity	Age, race
Multiple gestation	Assisted reproductive technologies

© 2025 Blauvelt CA et al. *JAMA Network Open.*

Assisted reproductive technologies	Age, private insurance
Early fetal growth restriction	Age, chronic hypertension, asthma, cardiovascular disease, diabetes, multiple gestation
Any COVID-19 vaccine	Private insurance
COVID-19 booster (2023-2024)	Private insurance
Influenza vaccine	Private insurance
Tdap vaccine	Private insurance

DAGitty Model Code:

```
dag {
"Asthma/Pulm disease" [exposure,pos="-1.441,-0.695"]
"Cardiovascular disease" [exposure,pos="-1.426,0.496"]
"Chronic HTN" [exposure,pos="-1.538,-0.159"]
"Early FGR" [exposure,pos="0.288,0.501"]
"Health seeking behavior" [latent,pos="0.376,-0.543"]
"Maternal age" [exposure,pos="-1.229,-1.633"]
"Multiple gestation" [exposure,pos="-0.336,0.720"]
"Non-English speaking" [exposure,pos="-0.357,-1.802"]
"Other maternal vaccines" [exposure,pos="0.376,0.105"]
"Private insurance" [exposure,pos="-1.477,-1.134"]
"Race/Ethnicity" [exposure,pos="-0.668,-1.847"]
"Socioeconomic status" [latent,pos="-0.538,-2.142"]
"Vaccine uptake" [outcome,pos="0.211,-1.261"]
ART [exposure,pos="0.026,0.748"]
Diabetes [exposure,pos="-1.010,0.672"]
Nulliparous [exposure,pos="-0.911,-1.757"]
Obesity [exposure,pos="-0.635,0.672"]
"Asthma/Pulm disease" -> "Early FGR"
"Asthma/Pulm disease" -> "Other maternal vaccines"
"Asthma/Pulm disease" -> "Vaccine uptake"
"Cardiovascular disease" -> "Early FGR"
"Cardiovascular disease" -> "Vaccine uptake"
"Cardiovascular disease" <-> "Chronic HTN"
"Chronic HTN" -> "Early FGR"
"Chronic HTN" -> "Vaccine uptake"
"Early FGR" -> "Vaccine uptake"
"Health seeking behavior" -> "Vaccine uptake"
"Health seeking behavior" <-> "Other maternal vaccines"
"Maternal age" -> "Cardiovascular disease"
"Maternal age" -> "Chronic HTN"
"Maternal age" -> "Early FGR"
"Maternal age" -> "Private insurance"
```

© 2025 Blauvelt CA et al. *JAMA Network Open.*

```
"Maternal age" -> "Vaccine uptake"
"Maternal age" -> ART
"Maternal age" -> Diabetes
"Maternal age" -> Nulliparous
"Maternal age" -> Obesity
"Multiple gestation" -> "Early FGR"
"Multiple gestation" -> "Vaccine uptake"
"Non-English speaking" -> "Socioeconomic status"
"Non-English speaking" -> "Vaccine uptake"
"Other maternal vaccines" -> "Vaccine uptake"
"Private insurance" -> "Other maternal vaccines"
"Private insurance" -> "Vaccine uptake"
"Private insurance" -> ART
"Race/Ethnicity" -> "Cardiovascular disease"
"Race/Ethnicity" -> "Chronic HTN"
"Race/Ethnicity" -> "Non-English speaking"
"Race/Ethnicity" -> "Socioeconomic status"
"Race/Ethnicity" -> "Vaccine uptake"
"Race/Ethnicity" -> Diabetes
"Race/Ethnicity" -> Obesity
"Socioeconomic status" -> "Private insurance"
"Socioeconomic status" -> "Vaccine uptake"
"Socioeconomic status" -> ART
ART -> "Early FGR"
ART -> "Multiple gestation"
ART -> "Vaccine uptake"
Diabetes -> "Cardiovascular disease"
Diabetes -> "Early FGR"
Diabetes -> "Vaccine uptake"
Nulliparous -> "Vaccine uptake"
Obesity -> "Asthma/Pulm disease"
Obesity -> "Cardiovascular disease"
Obesity -> "Chronic HTN"
Obesity -> "Vaccine uptake"
Obesity -> Diabetes
}
```

eFigure 3. Directed Acyclic Graph for Nirsevimab

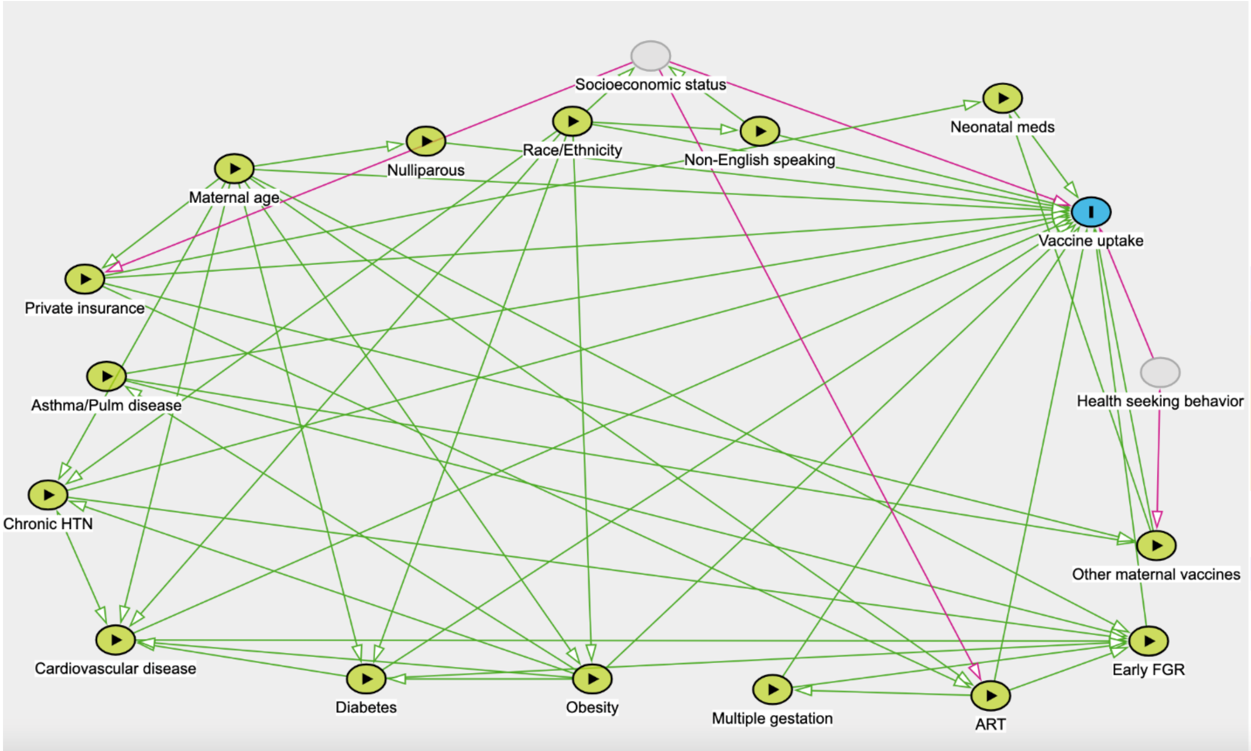

Covariate of interest	Secondary adjusted covariates
Age	None
Nulliparous	Age
Private insurance	Age, race, non-English language preference
Non-English language preference	Race
Race	None
Ethnicity	None
Asthma	Obesity
Other pulmonary disease	Obesity
Chronic hypertension	Age, race, diabetes, obesity, cardiovascular disease
Other cardiovascular disease	Age, race, chronic hypertension, diabetes, obesity
Pregestational diabetes	Age, race, obesity
Gestational diabetes	Age, race, obesity
Obesity	Age, race
Multiple gestation	Assisted reproductive technologies

© 2025 Blauvelt CA et al. *JAMA Network Open.*

Assisted reproductive technologies	Age, private insurance
Early fetal growth restriction	Age, chronic hypertension, asthma, cardiovascular disease, diabetes, multiple gestation
Any COVID-19 vaccine	Private insurance
COVID-19 booster (2023-2024)	Private insurance
Influenza vaccine	Private insurance
Tdap vaccine	Private insurance
Infant Hepatitis B vaccine	Private insurance, Tdap vaccine, Influenza vaccine
Erythromycin ointment	Private insurance, Tdap vaccine, Influenza vaccine

DAGitty Model Code:

```
dag {
"Asthma/Pulm disease" [exposure,pos="-1.441,-0.695"]
"Cardiovascular disease" [exposure,pos="-1.426,0.496"]
"Chronic HTN" [exposure,pos="-1.538,-0.159"]
"Early FGR" [exposure,pos="0.288,0.501"]
"Health seeking behavior" [latent,pos="0.307,-0.712"]
"Maternal age" [exposure,pos="-1.229,-1.633"]
"Multiple gestation" [exposure,pos="-0.336,0.720"]
"Neonatal meds" [exposure,pos="0.046,-1.951"]
"Non-English speaking" [exposure,pos="-0.357,-1.802"]
"Other maternal vaccines" [exposure,pos="0.301,0.070"]
"Private insurance" [exposure,pos="-1.477,-1.134"]
"Race/Ethnicity" [exposure,pos="-0.668,-1.847"]
"Socioeconomic status" [latent,pos="-0.538,-2.142"]
"Vaccine uptake" [outcome,pos="0.193,-1.437"]
ART [exposure,pos="0.026,0.748"]
Diabetes [exposure,pos="-1.010,0.672"]
Nulliparous [exposure,pos="-0.911,-1.757"]
Obesity [exposure,pos="-0.635,0.672"]
"Asthma/Pulm disease" -> "Early FGR"
"Asthma/Pulm disease" -> "Other maternal vaccines"
"Asthma/Pulm disease" -> "Vaccine uptake"
"Cardiovascular disease" -> "Early FGR"
"Cardiovascular disease" -> "Vaccine uptake"
"Cardiovascular disease" <-> "Chronic HTN"
"Chronic HTN" -> "Early FGR"
"Chronic HTN" -> "Vaccine uptake"
"Early FGR" -> "Vaccine uptake"
"Health seeking behavior" -> "Vaccine uptake"
```

```
"Health seeking behavior" <-> "Other maternal vaccines"
"Maternal age" -> "Cardiovascular disease"
"Maternal age" -> "Chronic HTN"
"Maternal age" -> "Early FGR"
"Maternal age" -> "Private insurance"
"Maternal age" -> "Vaccine uptake"
"Maternal age" -> ART
"Maternal age" -> Diabetes
"Maternal age" -> Nulliparous
"Maternal age" -> Obesity
"Multiple gestation" -> "Early FGR"
"Multiple gestation" -> "Vaccine uptake"
"Neonatal meds" -> "Vaccine uptake"
"Non-English speaking" -> "Socioeconomic status"
"Non-English speaking" -> "Vaccine uptake"
"Other maternal vaccines" -> "Neonatal meds"
"Other maternal vaccines" -> "Vaccine uptake"
"Private insurance" -> "Neonatal meds"
"Private insurance" -> "Other maternal vaccines"
"Private insurance" -> "Vaccine uptake"
"Private insurance" -> ART
"Race/Ethnicity" -> "Cardiovascular disease"
"Race/Ethnicity" -> "Chronic HTN"
"Race/Ethnicity" -> "Non-English speaking"
"Race/Ethnicity" -> "Socioeconomic status"
"Race/Ethnicity" -> "Vaccine uptake"
"Race/Ethnicity" -> Diabetes
"Race/Ethnicity" -> Obesity
"Socioeconomic status" -> "Private insurance"
"Socioeconomic status" -> "Vaccine uptake"
"Socioeconomic status" -> ART
ART -> "Early FGR"
ART -> "Multiple gestation"
ART -> "Vaccine uptake"
Diabetes -> "Cardiovascular disease"
Diabetes -> "Early FGR"
Diabetes -> "Vaccine uptake"
Nulliparous -> "Vaccine uptake"
Obesity -> "Asthma/Pulm disease"
Obesity -> "Cardiovascular disease"
Obesity -> "Chronic HTN"
Obesity -> "Vaccine uptake"
Obesity -> Diabetes
}
```

© 2025 Blauvelt CA et al. *JAMA Network Open.*

eTable 2. Characteristics Associated With RSVpreF Vaccination

Covariate	Vaccinated (n=414)	Unvaccinated (n=233)
Maternal age	35.3 (5.8)	33.4 (6.6)
Nulliparous	242 (58.5)	113 (48.5)
Private insurance	383 (92.5)	175 (75.1)
Non-English language preference	9 (2.2)	22 (9.4)
**Race**		
Asian	121 (29.2)	53 (22.8)
Black	21 (5.1)	31 (13.3)
White	180 (43.5)	70 (30.0)
Two or more races	21 (5.1)	11 (4.7)
Other or unknown race	73 (17.6)	68 (29.2)
**Ethnicity**		
Hispanic	56 (13.5)	62 (26.6)
Non-Hispanic	343 (82.9)	161 (69.1)
Unknown ethnicity	15 (3.6)	10 (4.3)
**Maternal medical conditions**		
Asthma	52 (12.6)	36 (15.5)
Other pulmonary disease	10 (2.4)	8 (3.4)
Chronic hypertension	33 (8.0)	25 (10.7)
Other cardiovascular disease	41 (9.9)	26 (11.2)
Pregestational diabetes	17 (4.1)	8 (3.4)
Gestational diabetes	56 (13.5)	31 (13.3)
Obesity	74 (17.9)	66 (28.3)
**Pregnancy conditions**		
Multiple gestation	4 (1.0)	8 (3.4)
Assisted reproductive technologies	60 (14.5)	29 (12.5)
Fetal growth restriction	34 (8.2)	16 (6.9)
Early fetal growth restriction	9 (2.2)	5 (2.2)
**Maternal vaccinations**		
Any COVID-19 vaccine	399 (96.4)	171 (73.4)
COVID-19 booster (2023-2024)	238 (57.5)	40 (17.2)
Influenza vaccine	374 (90.3)	117 (50.2)
Tdap vaccine	399 (96.4)	182 (78.1)

© 2025 Blauvelt CA et al. *JAMA Network Open.*

eTable 3. Characteristics Associated With Neonatal Nirsevimab Administration Prior to Hospital Discharge

**Covariate**	**Nirsevimab (n=183)**	**No Nirsevimab (n=78)**
Maternal age	33.2 (7.0)	33.4 (7.4)
Nulliparous	96 (52.5)	33 (42.3)
Private insurance	142 (77.6)	56 (71.8)
Non-English language preference	13 (7.1)	9 (11.5)
**Race**		
Asian	49 (26.8)	13 (16.7)
Black	23 (12.6)	8 (10.3)
White	56 (30.6)	25 (32.1)
Two or more races	9 (4.9)	2 (2.6)
Other or unknown race	47 (25.7)	30 (38.5)
**Ethnicity**		
Hispanic	45 (24.6)	25 (32.1)
Non-Hispanic	132 (72.1)	48 (61.6)
Unknown ethnicity	6 (3.3)	5 (6.4)
**Maternal medical conditions**		
Asthma	28 (15.3)	11 (14.1)
Other pulmonary disease	7 (3.8)	2 (2.6)
Chronic hypertension	25 (13.7)	6 (7.7)
Other cardiovascular disease	19 (10.4)	14 (18.0)
Pregestational diabetes	9 (4.9)	3 (3.9)
Gestational diabetes	26 (14.2)	8 (10.3)
Obesity	52 (28.4)	21 (26.9)
**Pregnancy conditions**		
Multiple gestation	5 (2.7)	4 (5.1)
Assisted reproductive technologies	26 (14.2)	7 (9.0)
Fetal growth restriction	12 (6.6)	6 (7.7)
Early fetal growth restriction	2 (1.1)	4 (5.1)
NICU admission	45 (24.6)	17 (21.8)
**Maternal vaccinations**		
Any COVID-19 vaccine	152 (83.1)	46 (59.0)
COVID-19 booster (2023-2024)	42 (23.0)	11 (14.1)

© 2025 Blauvelt CA et al. *JAMA Network Open.*

Influenza vaccine	109 (59.6)	33 (42.3)
Tdap vaccine	161 (88.0)	49 (62.8)
**Neonatal medications**		
Intramuscular Vitamin K	183 (100)	70 (89.7)
Hepatitis B vaccine	166 (90.7)	45 (57.7)
Erythromycin ophthalmic ointment	174 (95.1)	58 (74.4)

© 2025 Blauvelt CA et al. *JAMA Network Open.*

eTable 4. Multivariable Conditional Logistic Regression Analysis for Nested Case-Control Analysis With Preterm Birth as the Primary Outcome

**Covariate**	**Adjusted Odds Ratio (95% Confidence Interval)**
RSVpreF vaccination (binary)	1.03 (0.55 – 1.93)
Maternal age (continuous)	1.02 (0.95 – 1.09)
Nulliparous (binary)	0.80 (0.46 – 1.40)
Private insurance (binary)	0.76 (0.34 – 1.70)
Race (categorical)	
Asian	1.00 [Reference]
Black	1.60 (0.57 – 4.51)
White	1.18 (0.60 – 2.32)
Other	0.84 (0.36 – 2.00)
Hispanic ethnicity (binary)	1.40 (0.65 – 3.01)
Maternal cardiovascular disease (binary)	2.10 (1.01 – 4.34)
Maternal pregestational diabetes (binary)	3.07 (1.01 – 9.37)
Multiple gestation (binary)	33.05 (6.56 – 166.38)
Assisted reproductive technologies (binary)	0.84 (0.36 – 2.01)
Early fetal growth restriction (binary)	2.66 (0.70 – 10.16)
Tdap vaccine (binary)	0.79 (0.33 – 1.89)

Race and ethnicity data were obtained from the electronic medical record. The "Other" race category included self-reported Other race, Native American and Alaskan Native, Native Hawaiian or Pacific Islander, and Two or more races. Participants with chronic hypertension alone but not other cardiovascular diseases (i.e. arrhythmia, structural heart disease, cardiomyopathy) were not categorized as having maternal cardiovascular disease. Early fetal growth restriction includes cases diagnosed prior to 32 weeks' gestation. Tdap, Tetanus-Diphtheria-Pertussis.

© 2025 Blauvelt CA et al. *JAMA Network Open.*
