# Supplementary material for: Respiratory Syncytial Virus Vaccine and Nirsevimab Uptake Among Pregnant People and Their Neonates
Source: JAMA Netw Open. 2025 Feb 19;8(2):e2460735. doi: 10.1001/jamanetworkopen.2024.60735 (PMC11840647; doi:10.1001/jamanetworkopen.2024.60735)
Supplement: Supplement 2. — Data Sharing Statement [file jamanetwopen-e2460735-s002.pdf]

Data Sharing Statement

Blauvelt. Respiratory Syncytial Virus Vaccine and Nirsevimab Uptake Among Pregnant People and Their Neonates. *JAMA Netw Open*. Published February 19, 2025.
doi:10.1001/jamanetworkopen.2024.60735

Data
Data available: Yes
Data types: Deidentified participant data
How to access data: christine.blauvelt@ucsf.edu
When available: With publication

Supporting Documents
Document types: None

Additional Information
Who can access the data: We plan to share deidentified participant data after publication to researchers whose proposed use of the data has been approved.
Types of analyses: Any researchers whose proposed use of the data has been approved.
Mechanisms of data availability: With investigator support
